# Using biomarkers in acute medicine to prevent hearing loss: should this require specific consent?

**DOI:** 10.1136/medethics-2020-106106

**Published:** 2020-07-12

**Authors:** Peta Coulson-Smith, Anneke Lucassen

**Affiliations:** Clinical Ethics and Law Southampton (CELS), Faculty of Medicine, University of Southampton, Southampton, UK

**Keywords:** informed consent, genethics, genetic screening/testing, human tissue, minors/parental consent

## Introduction

In this round table response, we discuss some of the problems inherent in insisting on specific consent for an activity that needs to happen rapidly as part of a package of care. The Human Tissue Authority (the UK regulator for human tissue and organs) consider that specific consent is mandatory to assess which antibiotics are appropriate on the neonatal unit, but this insistence may actually limit the autonomy which consent aims to promote. While genetic testing to determine which child will react adversely to particular antibiotics has been available clinically for several years, the research proposed here is to assess whether improving the speed of testing allows decisions to be made before treatment starts. Insisting on specific consent before this activity can take place is likely to delay appropriate care in some cases.

## Package of care in neonatal intensive care

On admission to the neonatal unit, the unwell infant is routinely subjected to a range of investigations and treatments.

A person with parental responsibility will usually be asked to provide consent to undertake treatments for children who do not have capacity to give consent themselves.^1^ For the acutely unwell child, consent will usually be sought for a package of care: the urgent nature of neonatal medicine often requires clinicians to make many decisions without explicit consent. Doctors must always act in a child’s best interests: their decisions need to protect the health and well-being of a child and hold the child’s welfare paramount in accordance with the Children Act (1989) and General Medical Council (GMC) guidelines.^1 2^ Thus, for example, ventilation might be initiated, sedation provided, antibiotics commenced or diagnostic investigations requested without separate consent conversations or documentation of decisions on specific consent forms. McDermott *et al* propose to investigate a test to determine which children are likely to suffer complications from a treatment before that treatment is started. If such testing requires specific consent outside this overal package of care, then it may be more difficult to deliver appropriate care of the unwell infant.

## Point-of-care test

It has been known for some time that a mitochondrial variant (m.1555A>G) predisposes those who carry it to develop severe hearing loss if they receive aminoglycoside antibiotic treatment. The UK Genetic Testing Network - until recently the national advisory organisation for National Health Service clinical genetic testing services - offered this biomarker test as part of routine diagnostic care.^3^ However, such testing was usually too slow to influence acute treatment decisions since it took days/weeks to provide results.^4^ McDermott *et al* 5 have developed a point-of-care test (POCT) which speeds this process up considerably: a mouth brush can provide results within 30 minutes so that an alternative to aminoglycoside therapy can be considered for those who have the m.1555A>G variant.^5^ The Pharmacogenetics to Avoid Loss of Hearing (PALOH) trial is not investigating the genetic test itself, we already know this to be effective in predicting where aminoglycoside therapy should be avoided, but rather, whether the shorter time to obtain a result is helpful in the acute neonatal setting. Why then, would the Human Tissue Authority decide that the trial requires specific consent from a parent prior to performing the POCT, thus potentially requiring treatments to start before the result is known? We agree that such testing should not be done without consent, but we do not see the benefit in asking for specific consent where broad consent for a package of care in neonatal intensive care (including not providing treatments that are contraindicated) has been provided.

## The Human Tissue Act (2004)

Following the revelation that several English hospitals had retained patients’ body parts without the consent of their families, it became clear that existing law made no provisions to proscribe such behaviour, and such practices were considered to be unethical by the subsequent Kennedy and Redfern inquiries.^6^ A new Human Tissue Act (2004) was enacted in 2006, and provided clear hierarchies about who should give consent for storage of tissue from the deceased. The Act impinges very little on the everyday diagnosis, investigation and treatment of patients. These activities remain governed by the rules around obtaining a patient’s consent as provided in common law, the Mental Capacity Act (2005) and laid out in guidance by, among others, the GMC.^7^ This is why clinical testing for the m.1555A>G variant has been possible without engaging with the Human Tissue Act, however, research activity where tissue is held with an intention to analyse the DNA within it, does engage the Act. The Act was intended to regulate the retention of tissues and ensure that specific consent was obtained to retain tissues beyond their immediate diagnostic use. However, the stumbling block for the trial seems not to be an issue of consent to retain tissue, but rather to running the POCT without specific research consent.

Interestingly, situations where specific consent might not be possible was envisaged by legislators: Schedule 4 paragraph 9 of the Act lays down a route whereby testing could be done without specific consent.^8^ Although this route would not be suitable for the PALOH trial, it suggests legislators acknowledged that specific consent might not be possible in all situations and that this should not be a veto to activity where the benefits outweigh the harms. Yet the Human Tissue Authority's opinion is that the researchers that specific consent is a requirement. We query whether it is defensible not to provide a test to prevent hearing loss where this is possible?

## Opt-out consent

Since the PALOH trial is researching the effect of increasing the speed of an established clinical test, it has perhaps unwittingly entered a terrain not clearly governed by the Human Tissue Act—that of hybrid clinical/research activity.^9^ Yet once research ethics approval is sought, the Human Tissue Authority will assume that research activity rules should apply. What may further explain the resultant quagmire is that confusing terminology about consent has been applied by McDermott *et al*.^5^ Although ‘opt-out’ consent has been used in relation to organ donation it is, as the Information Commissioner outlines, an oxymoron.^10^ Consent should be a positive act, which, if omitted does not become consent by a later opportunity to opt-out. Perhaps the Human Tissue Authority considered that the PALOH trial was trying to proceed without any consent at all because of the use of this term? Yet, as outlined above, the opportunity for detailed, specific consent in acute care is often limited. The proposed POCT could be considered integral to the broad package of care offered to the acutely unwell neonate, for which broad parental consent is provided, or if unavailable, is done in the best interest of the child. Given that research is often seen as a luxury add-on to acute care, it is important to emphasise that what is being researched is whether a POCT can be incorporated into urgent care, not the genetic test itself. It is not clear to what extent the Human Tissue Authority decision was because the study proposed to research a genetic test,. Might the decision have been different if this was a biochemical assessment of a genetic marker? An abnormally high cholesterol level can equally indicate a genetic mutation.

## Conclusion

The research ethics approved PALOH trial tests the utility of an established clinical genetic test in an acute care setting, to see whether rapid results allow timely clinical decision making. Preventing such a trial on the grounds that it does not meet the requirements of the Human Tissue Act appears to go against the spirit of the Act and denies children a safe, evidence-based test that could prevent antibiotic-induced hearing loss.
